# Biased escorts: offspring sex, not relatedness explains alloparental care patterns in a cooperative breeder

**DOI:** 10.1098/rspb.2016.2384

**Published:** 2017-05-03

**Authors:** Emma I. K. Vitikainen, Harry H. Marshall, Faye J. Thompson, Jenni L. Sanderson, Matthew B. V. Bell, Jason S. Gilchrist, Sarah J. Hodge, Hazel J. Nichols, Michael A. Cant

**Affiliations:** 1 Centre for Ecology and Conservation, University of Exeter, Penryn Campus, Cornwall TR10 9FE, UK; 2 Institute of Evolutionary Biology, University of Edinburgh, Edinburgh, UK; 3 School of Applied Sciences, Edinburgh Napier University, Edinburgh, UK; 4 School of Natural Sciences and Psychology, Liverpool John Moores University, Liverpool L3 3AF, UK

**Keywords:** cooperative breeding, nepotism, helping, altruism, sex-biased care, alloparental care

## Abstract

Kin selection theory predicts that animals should direct costly care where inclusive fitness gains are highest. Individuals may achieve this by directing care at closer relatives, yet evidence for such discrimination in vertebrates is equivocal. We investigated patterns of cooperative care in banded mongooses, where communal litters are raised by adult ‘escorts’ who form exclusive caring relationships with individual pups. We found no evidence that escorts and pups assort by parentage or relatedness. However, the time males spent escorting increased with increasing relatedness to the other group members, and to the pup they had paired with. Thus, we found no effect of relatedness in partner choice, but (in males) increasing helping effort with relatedness once partner choices had been made. Unexpectedly, the results showed clear assortment by sex, with female carers being more likely to tend to female pups, and male carers to male pups. This sex-specific assortment in helping behaviour has potential lifelong impacts on individual development and may impact the future size and composition of natal groups and dispersing cohorts. Where relatedness between helpers and recipients is already high, individuals may be better off choosing partners using other predictors of the costs and benefits of cooperation, without the need for possibly costly within-group kin discrimination.

## 1. Introduction

Hamilton's rule [1] predicts when costly helping behaviour can evolve and provides a simple yet very broad framework for understanding evolution of altruism. Hamilton's rule predicts that, other things being equal, costly care should be directed to closer relatives. When relatedness to the potential recipients of helping behaviour varies, actors may be able to discriminate and direct help using cues that correlate with genetic relatedness [2]. Studies on cooperatively breeding animals have found evidence that choice of recipient or helping effort is positively correlated with kinship (e.g. white-fronted bee-eaters [3], Seychelles warblers [4] and long-tailed tits [5]), or, in a rare example, negatively correlated with kinship [6]. These studies support the notion that the capacity for kin discrimination is widespread among cooperatively breeding vertebrates.

In many contexts, however, kin discrimination is notably absent. In social insects, for example, individuals discriminate between nest-mates and non-nest-mates, but typically do not discriminate degrees of relatedness among nest-mates [7–9]. In many biparental and cooperative breeding systems, male parental investment is not related to paternity share [10–12]. A lack of kin discrimination in these contexts may reflect limitations imposed by the use of shared environmental or social cues of relatedness or parentage, or the costs of distinguishing degrees of relatedness when most interactions occur among kin of some kind [7]. However, it may also reflect selection on recipients to conceal identity. For example, being identified as highly related to a potential helper might simultaneously expose an individual to harmful or spiteful actions from non-related individuals [13]. Actors may thus be unable to discriminate even if it were in their interest to do so, if the benefits from directed care are outweighed by the costs of the recipient being discriminated against by other group members [14].

Hamilton's rule also predicts that helpers should be sensitive to variation in the benefits that different recipients stand to gain from a helpful act, and variation in personal fitness costs of allocating help to one individual over another. In mixed sex groups, these costs and benefits are likely to vary according to the sex of both actors and recipients. For example, helping may entail greater energetic costs, or greater risks, for one sex than another because of sex asymmetries in size, or physiological specialization (e.g. allosuckling; [15,16]). In males, helping may be incompatible with guarding fertile females, so they may experience greater opportunity costs by investing in care rather than in reproductive competition [17]. From the perspective of recipients, the benefit conferred by help may also vary systematically with sex. For example, in sexually dimorphic mammals offspring of the larger sex have higher energy requirements and may gain more (in terms of future reproductive success) from alloparental investment received [18–20].

Here, we ask whether variation in relatedness, or in ecological and social correlates of costs and benefits of helping, predicts patterns of alloparental care in cooperatively breeding banded mongooses (*Mungos mungo*). This species is ideal to test the factors that influence targeting of care because there are numerous helpers of each sex and numerous potential recipients of varying relatedness. Multiple females breed in each breeding attempt and give birth to a synchronous, communal litter, typically on the same day [21–23]. After these pups emerge from the den (at about one month of age), they are cared for by adult ‘escorts’ who form exclusive one-to-one helping relationships with particular pups, feeding and protecting them, and passing on foraging skills [24,25]. Both pups and adults exercise influence over the formation of escorting relationships: pups compete for certain escorts [26], and escorts recognize and single out their ‘own’ pup for preferential care [24,26,27]. While some pups in the litter are escorted by a particular escort every day, other pups receive much less care and must fend for themselves from an early age. Escorted pups receive more food, grow faster and are more likely to survive to independence than non-escorted pups [28].

We used a 15-year dataset on escorting in wild banded mongooses to test the relative influence of sex and relatedness on patterns of helping behaviour within and between litters. Specifically, we investigated the following three questions:

(1) Do patterns of relatedness predict allocation of care across litters?
(2) Does relatedness and/or sex predict which helpers and offspring form escorting associations, or the strength of these associations?
(3) What predicts variation in the total amount of escorting received by the offspring?

As we demonstrate, kinship has differing effects on helping effort and assortment, and our study reveals strong patterns of sex-specific helping. Our results also support earlier claims that mothers cannot recognize their own young in this communal breeding system.

## 2. Material and methods

### (a) Study species and population

Banded mongooses (*M. mungo*) are cooperatively breeding, diurnal carnivores in the family Herpestidae that are common in central and eastern parts of Africa. We conducted the study on a population of wild banded mongooses living on and around the Mweya peninsula of Queen Elizabeth National Park, Uganda (0°12′ S, 27°54′ E). For details of the field site and the population, see Cant *et al*. [29] and references therein. Reproduction is synchronized within social groups, and females give birth up to four times per year. The resulting mixed litter is reared communally by group members; both parents and non-breeding group members contribute to pup care [30].

All mongooses in our study population are individually marked using either unique hair-shave patterns or colour-coded collars, and most animals are habituated to close observation from at least 5 m and trained to step onto portable electronic scales to obtain weight measurements. One to two mongooses in each group are fitted with a radio collar weighing 26–30 g (Sirtrack Ltd, Havelock North, New Zealand) to allow the groups to be located. Pups were first captured at emergence from the den, at around three to four weeks of age, weighed, sexed and marked with permanent hair dye (see Jordan *et al*. [31] for further details of the trapping procedure). When individuals were first trapped, a 2 mm skin sample was taken for extraction of DNA, which was used to construct a pedigree for assigning parentage and calculation of pairwise relatedness values. The final pedigree used both Masterbayes 2.51 [32] and COLONY 2.0.5.7 [33] to infer parentage; 95% of parental assignments of individuals included in this study were made with greater than 90% confidence. For full details of DNA extraction, genotyping, parentage assignment and pedigree construction, see Sanderson *et al*. [34]. Weather data (rainfall) were collected by the Mweya weather station, and cumulative rainfall during the 30 days before the communal litter was born was used as a proxy of resource availability, as this has been found to affect competition and patterns of care in banded mongooses [35–37].

### (b) Quantifying escorting behaviour

**Figure 1.**
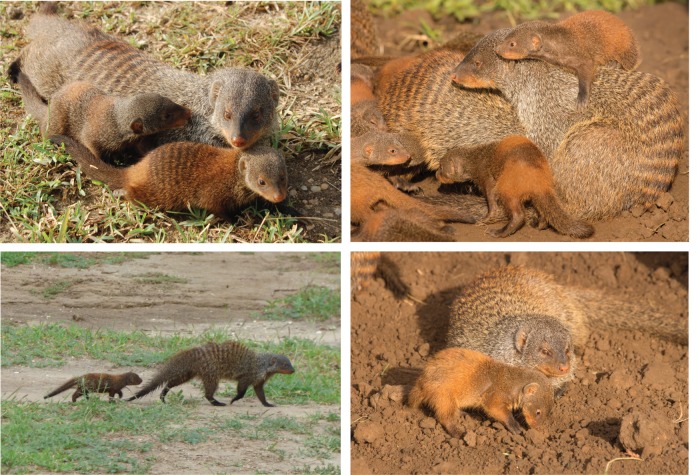
Escorts care for the pups carrying, feeding and grooming them.

Banded mongooses exhibit a conspicuous form of alloparental care termed ‘escorting’, whereby older individuals form one-on-one helping relationships with pups ([38] and references therein). Escorts stay close to their associated pup, feeding, carrying, grooming and protecting it from predators ([24], figure 1). Escorting starts when pups first emerge from the den at around four weeks old and continues until pups reach nutritional independence at the age of three months (the ‘escorting period’). We observed escorting behaviour in 143 communal litters in 12 social groups (mean group size 22 adult individuals; s.d. 7.3, range 7–37) that inhabited the study area between the years 2000 and 2015. During this escorting period, groups were visited on average 12 times for a minimum of 20 min (the duration of one focal observation session). Relatedness estimates and pup and escort weights were available for most but not all litters, so the actual sample size varied according to the available data and set of predictor variables included in each analysis, see details below and electronic supplementary material, tables S1–S3.

An adult individual was termed an escort if it associated closely with the focal pup, i.e. spent more than half of the 20 min observation session within 0.5 m of the focal pup (figure 1). Detailed focal observations of escorting effort of care show that pup provisioning rates are correlated with time spent in close association (*r* = 0.56, *n* = 129, *p* < 0.001 [39]), and escorts preferentially carry, groom and play with the pups they associate with [24,27,39]). Therefore, proportion of the sessions during which an adult was observed escorting a pup was used as a measure of its escorting effort, and the proportion of the observation sessions a pup was seen being escorted by an adult was used as a measure of escorting it received. Data on parentage and relatedness were not available at the time of behavioural data collection, and hence all the observations and scoring of escorting behaviour were done blind as to the relatedness and parentage between a pup and its escort.

### (c) Statistical analyses and model selection

We included as potential escorts all individuals that were present in a social group and over six months old at the time the litter was born, and escorting relationships between adults and pups, and the escorting effort given and received in those relationships were defined as above. We used generalized linear mixed models (GLMMs) with binomial error structure and logit link function, and social group, litter and individual identity were included as random effects to control for repeated sampling as detailed in the analysis sections below. Statistical analyses were done in R, v. 3.3.0 [40] and GLMM models fitted using R package lme4 [41]. Significance of terms was determined using likelihood ratio tests [41], and non-significant interactions were dropped from final models to allow significance testing of the main terms [42]. As female reproductive conflict increases with increasing numbers of females in the group [22,43] and male reproductive success similarly diminishes as a function of number of males in the group [44], we included the number of same-sex individuals in the group as proxy of the intensity of reproductive competition, but did not include total group size as this was highly collinear with both (number of females: *R*^2^ = 0.68, number of males: *R*^2^ = 0.95). Because males and females are known to differ in determinants of their helping effort [17,35], we carried out analyses 1 and 2 separately for males and females. Despite relatedness to the litter being correlated with parentage (*r*^2^ = 0.56), we included both in the models as it did not affect our results; for a more detailed discussion, see the electronic supplementary material. The level of correlation between other predictor variables in our models was below the level identified by Freckleton [45] as problematic for estimating linear models (all *r* < 0.4). The full analysis results including non-significant parameter estimates are presented in the electronic supplementary material, tables S1–S3.

We also used *t*-test to look at overall sex differences in pup weight, and Wilcoxon signed-rank tests to compare the total escorting effort female and male pups received. Average relatedness among realized and potential pup–escort pairs was compared using a *t*-test with 10 000 permutations, implemented in the R-package broman [46].

### (d) Question 1: Do patterns in relatedness predict allocation of care across litters?

First, we tested whether characteristics of the litter, social group or those of the potential escort at the time the litter was born predicted the escorting behaviour of adults in the group. Because different factors may predict whether an individual helps at all, and their total effort when they help, this analysis was conducted in two parts. First, we treated escort status as a binomial variable whether or not an individual was observed escorting in that litter at all. Second, we fitted the number of sessions an individual was observed escorting as the binomial response variable with the total number of observation sessions as the denominator, for individuals that had been escorting at least once in that litter. Splitting the analyses in this way also accounted for problems with zero-inflation in the data. Predictor variables in both analyses were rainfall during the previous month, weight and age of the potential escort and their interaction, number of pups in the litter, number of same-sex adults in the group, parentage (whether or not the focal individual was parent to any pups in that litter), the focal individual's average relatedness to pups in the litter and the focal individual's average relatedness to adults in the group. Both analyses included individual, litter and social group as random effects.

### (e) Question 2: Does relatedness and/or sex predict which helpers and pups form escorting associations, or the strength of these associations?

We then looked at pairwise interactions between pups and escorts within-litter. Similar to analysis 1, this analysis was done in two parts, as different factors may predict which pups an adult associates with, and how much care is given when they do. First, we conducted a binomial GLMM looking at whether an escort associated with a given pup (0/1). For this all potential pairs of pups and escorts were constructed, so that all individuals that were observed escorting at least once in a given litter were included as potential escorts for all the pups in that litter. Predictor variables were sex of the pup, pup weight, parentage and relatedness between the adult and the pup, and to investigate whether escorting might be contingent on within-sex competition, we also included as a predictor the interaction between the number of same-sex adults and the sex of the pup. Second, among observed pup–escort pairs, we used a binomial GLMM with the proportion of observation sessions the focal pup was being escorted by the focal adult as the binomial response variable, using the same set of predictor variables as above. While most of the relationships are dyadic in nature, an adult can sometimes escort multiple pups in a litter and a pup may have multiple escorts. To account for this, we included both pup and escort identity, as well as litter and social group, as random effects in these analyses.

### (f) Question 3: What predicts variation in the total amount of care received by the offspring?

Finally, we looked at escorting relationships from the pup perspective, with the analysis split as above. First, we looked at whether or not a pup associated with any escort (0/1), with pup sex, weight, litter size and their two-way interactions as predictors, and litter and social group as random effects (not individual, as each pup was only included once in this dataset). Second, we looked at predictors of the amount of care those pups received that had an escort (proportion of observation sessions they were escorted) with pup weight, pup sex, litter size, sex of the escort, parentage and relatedness between pup and the escort as covariates. In cases where the pup had multiple escorts, we included the characteristics of the adult that provided most care. In this analysis, escort identity, litter and social group were included as random effects.

## 3. Results

### (a) Question 1: Do patterns in relatedness predict allocation of care across litters?

**Figure 2.**
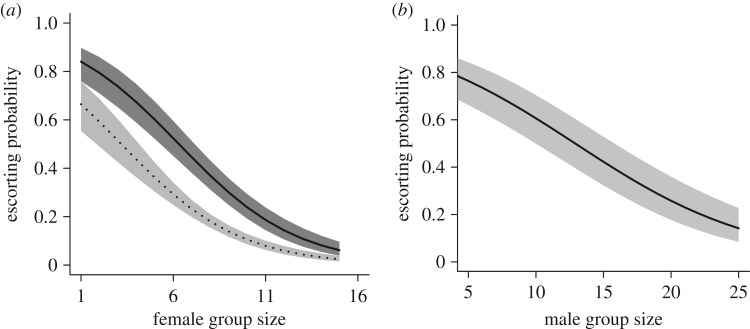
Effects of group size and parentage on patterns of escorting in banded mongooses. (*a*) Across litters, females were more likely to escort when the litter contained some of their own young, and their probability of escorting decreased with the number of females in the social group (mothers, dark-grey shaded area and solid line; non-mothers, light-grey area and dotted line). (*b*) Males were less likely to escort when there were more males in the social group, but whether they sired pups did not predict whether they escorted in a given litter. Lines represent model predictions ± s.e. from binomial GLMMs after controlling for random effects of individual, litter and social group.

Neither relatedness to the litter (after controlling for the effect of parentage; see electronic supplementary material) nor relatedness to other group members predicted the probability that a female escorted in a given litter (relatedness to the litter: 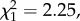 *p* *=* 0.133; relatedness to the group: 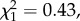 *p* *=* 0.511). Females were more likely to escort when they had mothered pups in the current litter (*β* ± s.e.: 0.98 ± 0.30, 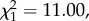 *p* < 0.001). In addition, the probability that each female would escort in the current litter declined with the number of adult females in the group (*β* ± s.e.: −0.31 ± 0.06, 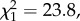 *p* < 0.001, figure 2*a*), and increased with increasing litter size (litter size: *β* ± s.e. = 0.14 ± 0.04, 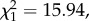 *p* < 0.001). The effect of weight on escorting probability was contingent on age, with the probability of escorting declining with age in heavier but not in lighter females (interaction weight × age: 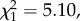 *p* *=* 0.024; electronic supplementary material, table S1*a* and figure S1*a*). Among those females that escorted, the total individual escorting effort per litter (proportion of sessions seen escorting) decreased as the number of females in the group increased (*β* ± s.e. = −0.068 ± 0.03, 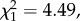 *p* = 0.024), but other terms had no effect on the total amount of help escorting females allocated to a litter; see electronic supplementary material, table S1 for full results.

For males, neither parentage nor relatedness predicted the probability that a male escorted in a given litter (parentage: 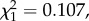 *p* = 0.744; relatedness to litter: 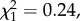 *p* *=* 0.826; relatedness to adults: 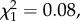 *p* *=* 0.775). Like females, the probability of a male escorting in a given litter declined with increasing number of adult males in the group (*β* ± s.e.: = −0.15 ± 0.03, 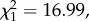 *p* *<* 0.001, figure 2*b*) and increased with increasing litter size (*β* ± s.e. = 0.21 ± 0.034, 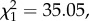 *p* *<* 0.001). The probability of escorting also increased with increasing rainfall (*β* ± s.e. = 0.27 ± 0.13, 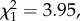 *p* = 0.047). Among those males that did escort, total escorting effort (proportion of sessions seen escorting) increased with relatedness to other adults in the group (*β* ± s.e. = 1.80 ± 0.65, 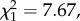 *p* *=* 0.006; electronic supplementary material, figure S2), but not with relatedness to the litter (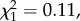 *p* *=* 0.74). The escorting effort of males also declined with increasing number of adult males in the group (*β* ± s.e. = −0.036 ± 0.01, 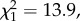 *p* *<* 0.001), but litter size had no effect on the total escorting effort (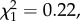 *p* = 0.637). As in females, the effect of weight on escorting depended on age, with both the likelihood of escorting and escorting effort decreasing with age in heavy but not in light individuals (electronic supplementary material, table S1 and figure S1*b*). The escorting effort of males was not predicted by rainfall (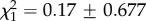).

### (b) Question 2: Does relatedness and/or sex predict which helpers and offspring form escorting associations, or the strength of these associations?

Pairwise relatedness did not predict associations in female escorts (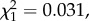 *p* *=* 0.859) nor in male escorts (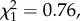 *p* *=* 0.383), and neither did parentage (females: 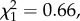 *p* = 0.414; males: 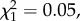 *p* = 0.832). Average relatedness between realized pup–escort pairs did not differ from that between pups and all potential escorts (average ± s.d.: realized pairs: *R* = 0.211 ± 0.169, all potential pairs: *R* = 0.208 ± 0.171, permutation test: *t* = 0.172, *p* = 0.87). Parentage did not predict the amount of care escorts gave to a particular pup (female escorts: 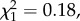 *p* = 0.675; male escorts: 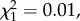 *p* = 0.934). For males but not females, escorting effort in escort–pup pairs increased with increasing dyadic relatedness between escort and pup (*β* ± s.e. = 0.83 ± 0.32, 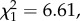 *p* = 0.010, females: 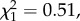 *p* = 0.476).

**Figure 3.**
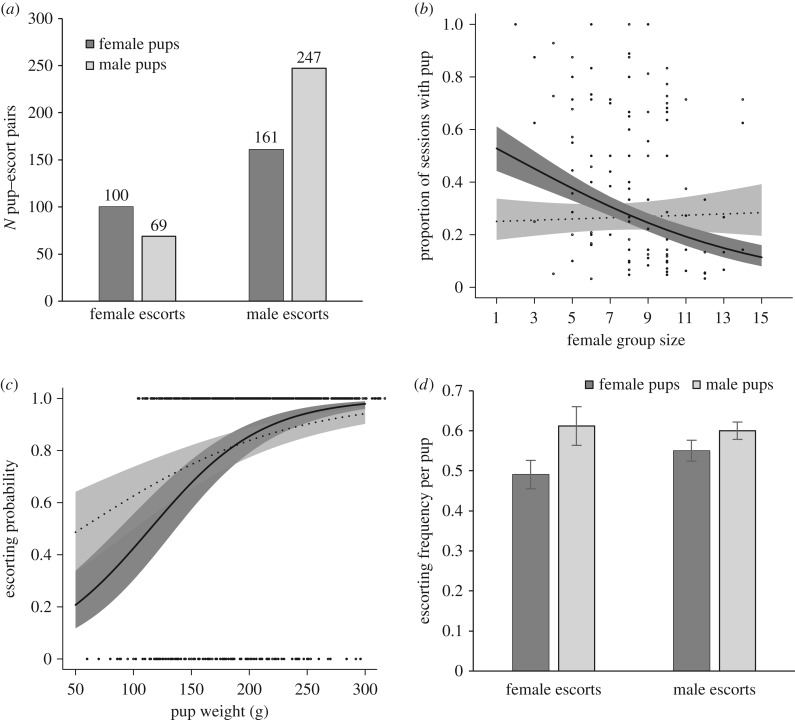
Sex differences in patterns of care. (*a*) Female escorts were more likely to pair with female pups (dark-grey bars) and male escorts with male pups (light-grey bars). Numbers are counts of observed escorting relationships. (*b*) Female escorts allocated more care to female pups (dark grey area, solid line) when compared with male pups (light-grey area, dotted line) when the adult female group size was low. Lines in (*b*) and (*c*) represent GLMM model predictions ± s.e. and dots present raw data, see electronic supplementary material, tables S1–S3 for full results. (*c*) Heavier pups were more likely to be escorted, and the effect of weight was more pronounced in female pups (dark-grey area, solid line) than in male pups (grey area, dotted line). (*d*) Overall, male pups received more care in existing escorting relationships than female pups, both from male and female escorts.

The sex of both the escort and the pup predicted patterns of dyadic association (figure 3*a*). Female escorts were more likely to pair with a female pup (59% of female escorts paired with a female pup versus 41% with a male pup; pup sex [M]: *β* ± s.e. = −0.43 ± 0.19, 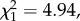 *p* *=* 0.023, figure 3*a*) and allocated more care to female pups when there were fewer females in the groups (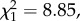 *p* *=* 0.003, figure 3*b*). Other factors did not predict dyadic association or the amount of care provided by females, see electronic supplementary material, table S2, for full results. Similarly, males were more likely to associate with male pups (61% of male escorts paired with male pups versus 39% with female pups; pup sex [M]: *β* ± s.e. = 0.40 ± 0.12, 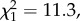 *p* < 0.001, figure 3*a*), but provided less care when there were more males in the group irrespective of the sex of the pup (*β* ± s.e. = +0.033 ± 0.01, 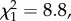 *p* *=* 0.003; for full results, see electronic supplementary material, table S2).

### (c) Question 3: What predicts variation in the total amount of care received by the offspring?

Compared with female pups, male pups received more care from helpers (proportion of observation sessions being escorted for all escorted and non-escorted pups: mean ± s.e.: males 0.478 ± 0.017 versus females 0.425 ± 0.019, Wilcoxon rank-sign test: *N* = 726, *W* = 100150, *p* = 0.034). Male pups were also slightly heavier at emergence than females (204.7 ± 3.1 versus 195.7 ± 3.1 g, *t*_724_ = 2.07, *p* *=* 0.039).

The effect of pup weight on its probability of being escorted depended on sex of the pup: larger pups more likely to be escorted, and the probability of escorting increased more steeply with weight in female pups (weight × pup sex: 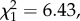 *p* *=* 0.042, figure 3*c*). When escorted, larger pups also received more care (*β* ± s.e. = 0.063 ± 0.031, 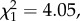 *p* *=* 0.044) than did male pups (*β* ± s.e. = 0.112 ± 0.057, 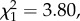 *p* *=* 0.051; figure 3*d*). Pups in larger litters were no more or less likely to be escorted (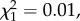 *p* *=* 0.907) but received less care when escorted (*β* ± s.e. = −0.024 ± 0.01, 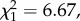 *p* *=* 0.009). Neither the relatedness between the pup and the escort providing most care, parentage, nor sex of the escort predicted the total amount of care that the escorted pups received (relatedness: 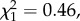 *p* *=* 0.496; escort sex: 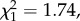 *p* *=* 0.187, parentage: 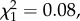 *p* *=* 0.775).

## 4. Discussion

Much research has focused on the influence of relatedness on selection for helping behaviour [47], but why individuals might conceal identity or avoid discrimination within groups is a much less explored topic, particularly in vertebrates. The naive prediction from Hamilton's rule is that care should be directed at closer relatives, but this ignores the problems that being able to discriminate creates, both at the individual and at the group level. Nepotism can be disruptive to the group and lead to selection against the very recognition cues that form the basis of such discrimination [7,9]. More pressingly, for the recipient to identify themself as highly related to some group members also comes at the cost of revealing themself as less related to others, exposing them to negative discrimination and spite [14,48]. Where the average relatedness between helpers and helpees is high anyway, such costs may override any benefit, making returns from discrimination marginal at best [7,12]. In the banded mongoose, the average relatedness between potential helpers and recipients was close to that between half-siblings (*R* ± s.d.: 0.21 ± 0.17). In such systems, individuals may be better off adjusting their behaviour according to other predictors of costs and benefits of cooperation, without the need for possibly costly within-group kin discrimination, and this is indeed what we find in the banded mongoose.

In the banded mongoose, a striking feature of their cooperative behaviour is the formation of one-to-one caring relationships termed ‘escorting’ [24]. Earlier studies have suggested that pups have an active role in establishing relationships with particular escorts [26,27,49], implying that the escort–pup relationship is not solely the result of choices made by the helper. However, previously, we have been unable to exclude the possibility that escorts were typically the parents of the pups they care for, bringing into question whether adults that engage in this behaviour should be termed helpers at all [50]. This study shows that escorts do indeed care for pups that are not their own offspring, and that, despite the presence of high-relatedness offspring within the communal litter, neither males nor females preferentially form pairwise associations with pups that are more related to them. Although females are more likely to escort when the current litter contains some of their own young, they do not preferentially pair with their own offspring, supporting previous claims that mothers do not or cannot discriminate their own young in the communal litter [21,38,51]. Moreover, neither pairwise relatedness nor parentage predicts the amount of care females allocated to an individual pup. The lack of kin discrimination by females is perhaps surprising given that escorting boosts the survival and growth of pups [26,28]. However, in banded mongooses, the potential costs of nepotistic discrimination may be particularly high because within-group infanticide is common [52]. Any pup advertising its close relatedness to a particular female (or, potentially, male) could be targeted by others and could also lose out on allosuckling by other females, even if not directly aggressed [53].

In males, neither paternity nor relatedness to the pups predicted patterns of assortment in escort–pup relationships. Nor did relatedness predict male escorting effort across litters. However, we did find two correlations between relatedness and patterns of male helping. First, across litters, males increased the time spent escorting when they were more closely related on average to the rest of the group. Second, within pup–escort pairs, more related dyads spent more time together. These results might suggest kin discrimination by males. However, these patterns could also arise as a result of other factors that are correlated with relatedness. For example, there may be subtle similarities in genetically heritable foraging preferences or character traits, such as preference of closed versus open habitat, or boldness and shyness, that could explain why more related partners spend more time together. There may also be subtle effects of group size on the observed relationships between escorts and pups. In small groups, in which relatedness is high, pups are particularly valuable in terms of group recruitment, and all adults may be more attentive escorts. Without cross-fostering experiments to manipulate which pups pair with which escorts, or experimental manipulation of group size, we are currently unable to fully understand the causality of the relationship between relatedness and helping effort in males.

We did find strong discrimination based on sex of the recipient. Both males and females were more likely to pair with a pup of their own sex and reduced their overall helping effort in response to increasing number of same-sex adults in the group. As group size was highly correlated with numbers of both adult males and females, individuals may simply reduce their contribution to care as there are more helpers present. However, females also provided more care to female pups when adult female numbers were low, which implies that within-sex cooperation and competition may be driving the preferential direction of help to the same sex. For female banded mongooses, there appears to be an optimal group size that maximizes their reproductive success [52]. Females are evicted in same-sex cohorts when the number of breeding females grows large [53–55], and patterns of dispersal and eviction may therefore create incentives for female adults to adjust care given towards female pups depending on the competitive environment. Males may also have an incentive to target care towards other males, since males may be particularly important in defending the territory against neighbouring groups and evicted cohorts of males that attempt to take over and supplant existing males [29].

Sex bias in care has been observed in many biparental birds, as well as other cooperatively breeding mammals, with varying direction of bias and consequences for the offspring. For example, in the toc-toc (*Foudia sechellarum*), the brood is divided by sex post-fledging between the mother and the father [56] with no overall differences between the sexes in the amount of care. In zebra finches, mothers preferentially provision sons over daughters, while fathers show no bias, and sons receive more food than daughters overall [57]. In social animals in particular, offspring of the same sex may be reproductive competitors or future helpers/soldiers, and depending on the system, helpers might prefer to raise offspring of the same or different sex. For example, in the cooperatively breeding arabian babblers (*Turdoides squamiceps*), helpers invest in offspring of the opposite sex in order to avoid competition [58], as do spotted hyaenas (*Crocuta crocuta*), where males associate more with daughters, than with sons [59]. Preferential helping of the same sex has been previously observed in the cooperatively breeding meerkat (*Suricatta suricata*). Similar to the banded mongoose, meerkat female helpers preferentially feed female pups, but males show no bias [60]. Females also provide more help than males. These patterns of care may be explained by sex differences in dispersal and the benefits of philopatry. In meerkats, males are the dispersing sex, and hence benefit less from any group augmentation benefits of helping compared with females. This explanation fits with our findings in the banded mongoose, where both sexes remain in their natal group, and are also more likely to pair up with a pup of the same sex.

Another explanation for the sex bias in caring relationships observed here is that the competitive ability of the pups may be driving the association. Male banded mongooses are more likely to be escorts than females, and they also provide more care. Larger pups were more likely to be escorted and received more care, despite the caring effort of individual helpers not being correlated with pup size. As male pups were on average slightly larger than female pups, they also received more care overall, with the total amount of escorting care received increasing more steeply with size in female than in male pups. This result suggests that bigger pups may be able to secure the best helpers, which often are young males. Our results are consistent with previous findings that escort–pup associations may arise from competitive differences between pups [24,26]. They also highlight our recent findings that mothers invest in larger fetuses when post-natal reproductive competition is likely to be intense [43]. Priming offspring early in development to compete for escorts may be a good competitive strategy when there is little or no opportunity to discriminate and direct nepotism towards one's own offspring after birth.

To conclude, we find that partner choice in the banded mongoose escorting system is the result of sex-specific association but not fine-grained discrimination of dyadic relatedness. In this system, advertisement of relatedness or identity is likely to involve costs in terms of exposure to aggression or infanticide, which far outweigh any potential benefits of nepotistic assortment. By contrast, the sex of offspring is a conspicuous and unchanging trait, which may act as predictor of direct fitness returns of investment for male versus female helpers. Since escorts boost offspring survival [28], and pass on foraging traditions to the pups in their care [25], sex-biased patterns of assortment may have lasting impacts on sex-specific behaviour, group composition and dispersal and breeding success of same-sex cohorts. Recent demographic models of social evolution have highlighted the impact of demography on selection for helping and harming (reviewed in [61]). Our findings raise the possibility that within-group assortative patterns of helping and harming can in turn feed up to influence demography in natural populations.

## Supplementary Material

Supplementary Information for Vitikainen et al. 2017, pup escorting in the banded mongoose

## Data accessibility

The data supporting the analyses are deposited online on Figshare at 10.6084/m9.figshare.4557136.
