# Supplementary material for: Biased escorts: offspring sex, not relatedness explains alloparental care patterns in a cooperative breeder
Source: Proc Biol Sci. 2017 May 3;284(1854):20162384. doi: 10.1098/rspb.2016.2384 (PMC5443930; doi:10.1098/rspb.2016.2384)

SUPPLEMENTARY ONLINE MATERIAL

Biased escorts: offspring sex, not relatedness explains alloparental care patterns in a cooperative breeder

Emma I. K. Vitikainen, Harry H. Marshall, Faye J. Thompson, Jenni L. Sanderson, Matthew B. V. Bell, Jason S. Gilchrist, Sarah J. Hodge, Hazel. J. Nichols & Michael A. Cant

Proceedings of The Royal Society B: Biological Sciences
DOI: 10.1098/rspb.2016-2384

SUPPLEMENTARY METHODS

Disentangling the effects of relatedness and parentage

In the banded mongoose, the potential caregivers termed escorts include the parents of the pups as well as all adults in the group. We were interested in determining whether relatedness and/or parentage best explained pairing between pups and helpers, and what determined the care given in these relationships. Relatedness to the litter was correlated with parentage (r^2=0.56), so we initially ran the models leaving relatedness and parentage out in turn. As significance of the other terms in the models was not contingent on whether parentage, relatedness, or both were included as predictors, this collinearity did not cause problems in terms of model performance, and we report the results from the full models containing both terms.

The collinearity between parentage and relatedness also has implications on interpretation of the terms themselves. Parent and offspring pairs are by definition highly related, but not all related pairs are between parents and offspring, which means that the effects can be teased apart.
One way to look at this is that since parentage and relatedness are highly correlated, including both as predictors controls for the effect of parentage on relatedness (Freckleton 2002) and is therefore a way to test whether there are effects of relatedness **over and above those caused by parentage.** This interpretation is corroborated with example using model described in Table 1B, predictors of female escorting behaviour in a given litter. When including both parentage and mean relatedness to the litter, only parentage effect comes out as significant (Table 1a) however, if parentage is left out of the model, relatedness to the litter becomes significant ($\beta \pm$ SE = 3.01 $\pm$ 1.08, χ^2_1 = 8.01, p = 0.005).

References:

Freckleton, R.P., 2002. On the Misuse of Residuals in Ecology: Regression of Residuals vs. Multiple Regression. *Journal of Animal Ecology*, 71(3), pp.542–545.

Cant, M. A., Vitikainen, E. & Nichols, H. J. 2013 Demography and Social Evolution of Banded Mongooses. Adv. Study Behav. 45, 407–445.(doi:10.1016/B978-0-12-407186-5.00006-9)

Nichols, H. J., Amos, W., Cant, M. A., Bell, M. B. V. & Hodge, S. J. 2010 Top males gain high reproductive success by guarding more successful females in a cooperatively breeding mongoose. *Anim. Behav.* **80**, 649–657. (doi:10.1016/j.anbehav.2010.06.025)

SUPPLEMENTARY TABLES

Table S1: Predictors of escorting behaviour (A) and effort (B) between litters in male and female banded mongooses, results from a binomial GLMM including individual, litter, and social group as random effects. Nonsignificant interactions (p > 0.05) were dropped to allow significance testing of main terms, but model was not simplified further; statistically significant estimates are reported in bold. To improve model fit, weight was standardised by subtracting the mean and dividing by standard deviation.

(A) Adult is observed escorting in current litter (0/1)

	Females			***Males***		
**Fixed effects**	β ± SE	χ^2_1	*P*	β ± SE	χ^2_1	*P*
Parentage (0/1)	**0.98 ± 0.30**	**11.00**	**<0.001**	0.11 ± 0.32	0.107	0.744
Mean relatedness to litter	1.71 ± 1.14	2.25	0.133	0.24 ± 1.10	0.048	0.826
* Mean relatedness to litter when parentage dropped	**3.01 ± 1.08**	**8.01**	**0.005**	-0.44 ± 0.37	1.38	0.240
Mean relatedness to adults	1.14 ± 1.72	0.43	0.511	-0.53 ±1.91	0.082	0.775
N emerged pups	**0.14 ± 0.04**	**15.94**	**<0.001**	**0.21 ± 0.034**	**35.05**	**<0.001**
Rainfall past 30 days	-0.19 ± 0.14	1.86	0.173	**0.27 ± 0.13**	**3.95**	**0.047**
N same-sex adults	**-0.31 ± 0.06**	**23.81**	**<0.001**	**-0.15 ± 0.03**	**16.99**	**<0.001**
Weight (standardized)	**0.98 ± 0.30**	-	-	**2.08 ± 0.28**	-	-
Age (months)	**-0.14 ± 0.09**	**-**	**-**	**-0.13 ± 0.08**	-	-
**Weight * Age**	**-0.24 ± 0.11**	**5.10**	**0.024**	**-0.26 ± 0.07**	**14.67**	**<0.001**
Number of observations	individuals 135: litters: 110; packs: 9			Individuals: 188; litters: 110, packs: 8		

(B) Escorting effort in current litter (proportion of sessions escorting observed)

	Females			***Males***		
**Fixed effects**	**β ± SE**	χ^2_1	***P***	**β ± SE**	χ^2_1	***P***
Parentage (0/1)	0.010 ± 0.14	0.006	0.941	-0.078 ± 0.10	0.63	0.427
Mean relatedness to litter	0.065 ± 0.68	0.016	0.923	0.13 ± 0.40	0.11	0.744
**Mean relatedness to adults**	1.12 ± 0.90	0.21	0.219	**1.80 ± 0.65**	**7.67**	**0.006**
N emerged pups	-0.034 ± 0.02	2.97	0.084	0.005 ± 0.01	0.22	0.637
Rainfall past 30 days	-0.077 ± 0.07	1.13	0.287	0.018 ± 0.04	0.17	0.677
**N same sex adults**	**-0.068 ± 0.03**	**4.49**	**0.034**	**-0.036 ± 0.01**	**13.9**	**<0.001**
Weight (standardized)	-0.008 ± 0.12	0.004	0.951	0.16 ± 0.099	-	-
Age (months)	-0.026 ± 0.05	0.30	0.584	0.018 ± 0.03	-	-
**Weight * Age**	-0.039 ± 0.07	0.31	0.581	**-0.058 ± 0.027**	**4.77**	**0.029**
Number of observations	Individuals: 67; litters: 68; packs: 8			Individuals: 117; litters: 94, packs: 7		

Table 2: Predictors of pairwise bonds between female and male escorts and pups (A) and the amount of help male and female escorts allocated to each pup in existing pup-escort pairs (B). For factors, parameter estimates are given in reference to the value in [brackets]. Binomial GLMMs were fitted with pup identity, escort identity, litter, and social group as random effects. Nonsignificant ($p<0.05$) interactions were dropped to allow testing of main effects, but the model was not simplified further.

(A) Adult is escorting the focal pup (0/1)

	Females			***Males***		
**Fixed effects**	**β ± SE**	χ^2_1	***P***	**β ± SE**	χ^2_1	***P***
Parentage	0.28 ± 0.34	0.66	0.414	-0.067 ± 0.32	0.05	0.832
Relatedness to the pup	0.12 ± 0.67	0.031	0.859	-0.39 ± 0.45	0.76	0.383
**Sex of pup [Male]**	**-0.43 ± 0.19**	**4.94**	**0.023***	**0.400 ± 0.12**	**11.3**	**<0.001**
Pup weight	0.07 ± 0.096	0.56	0.453	0.120 ± 0.063	3.39	0.065
**N same sex individuals**	-0.005 ± 0.038	0.018	0.893	**-0.033 ± 0.014**	**3.91**	**0.048**
Sex of pup * N same sex individuals	0.021 ± 0.079	0.069	0.792	-0.037 ± 0.023	2.64	0.104
Number of observations	Pups: 402; escorts: 82; litters: 76; packs: 8			Pups: 528; escorts: 145; litters: 111; packs: 8		

(B) Escorting effort (proportion of sessions escorting the focal pup

	Females			***Males***		
**Fixed effects**	**β ± SE**	χ^2_1	***P***	**β ± SE**	χ^2_1	***P***
Parentage	0.099 ± 0.24	0.18	0.675	-0.017 ± 0.22	0.01	0.934
Relatedness to the pup	-0.34 ± 0.47	0.51	0.476	**0.83 ± 0.32**	**6.61**	**0.010**
Sex of pup [Male]	**-1.47 ± 0.49**	-	-	0.087 ± 0.086	10.1	0.315
Pup weight	0.046 ± 0.076	0.35	0.552	0.08 ± 0.045	3.27	0.071
N same sex individuals	**-0.15 ± 0.047**	-	-	**-0.033 ± 0.01**	**8.80**	**0.003**
Sex of pup * N same sex individuals	**0.17 ± 0.056**	**8.85**	**0.003**	-0.017 ± 0.017	1.10	0.295
Number of observations	Pups: 121, escorts: 71; litters: 62; packs: 8			Pups: 302, escorts: 131; litters: 104; packs: 8		

Table S3: Predictors of (A) whether a pup was escorted by any adult(s) and (B) the total amount of help pup received from escorts, results from a binomial GLMM with escort identity, litter, and social group as random factors. For factors, parameter estimates are given in reference to the value in [brackets]. Nonsignificant interactions were dropped to allow significance testing of main terms, but models not simplified further. To improve model fit, pup weight was standardised by subtracting the mean and dividing by standard deviation.

(A) Probability of pup being escorted by an adult

Fixed effects	β ± SE	χ^2_1	*P*
Sex of pup [Male]	-0.11 ± 0.25	-	-
Pup weight (standardized)	1.23 ± 0.25	-	-
N emerged pups	-0.005 ± 0.041	0.01	0.907
**Sex of pup * pup weight**	**-0.56 ± 0.28**	**6.43**	**0.042**
Sex of pup * N emerged pups	0.061 ± 0.050	1.52	0.217
Litter size * pup weight	0.030 ± 0.033	0.787	0.375
Number of observations	Pups: 726, litters: 143, packs: 12		

(B) Total help received (proportion of sessions pup was escorted)

Fixed effects	β ± SE	χ^2_1	*P*
Relatedness to the escort	0.148 ± 0.217	0.46	0.496
Parentage	0.038 ± 0.132	0.08	0.775
Sex of escort [Male]	0.102 ± 0.077	1.74	0.187
**Sex of pup [Male]**	**0.112 ± 0.057**	**3.80**	**0.051**
**Pup weight (standardized)**	**0.063 ± 0.031**	**4.05**	**0.044**
**N emerged pups**	**-0.024 ± 0.010**	**6.67**	**0.009**
Sex of escort * pup weight	0.036 ± 0.074	0.24	0.621
Sex of escort * sex of pup	-0.169 ± 0.133	1.63	0.202
Sex of pup * N emerged pups	-0.016 ± 0.011	2.05	0.152
Number of observations	N pups: 373, escorts: 179, litters: 111, packs: 9		

SUPPLEMENTARY FIGURES

Figure S1.

Probability of escorting in a given litter showed an interaction between age and weight in both females (A) and males (B). Among young males and females, those that were heavy for their age escorted more than light individuals, and the pattern reversed as the individuals got older, with light individuals providing more care. This may reflect a trade-off between investment in care and reproduction, with heavy individuals specialising in direct fitness, and light individuals that are less likely to reproduce, in indirect fitness. Interestingly, the age at which the heaviest individuals start to invest less in escorting, is lower for females than it is for males, which may reflect life-history differences between males and females, with females reproducing earlier and males 'queuing' in order to reproduce (e.g. Cant et al. 2013, Nichols et al. 2010). Lines and shaded areas represent model predictions from binomial GLMMs ± SE. Weight was treated as a continuous variable in the model, but the model predictions are plotted for illustrative purposes for heavy individuals (75% quantile; dark shaded area, solid line) and light individuals (25% quantile, light shaded area, dotted line). Dots are raw data points.

(A)

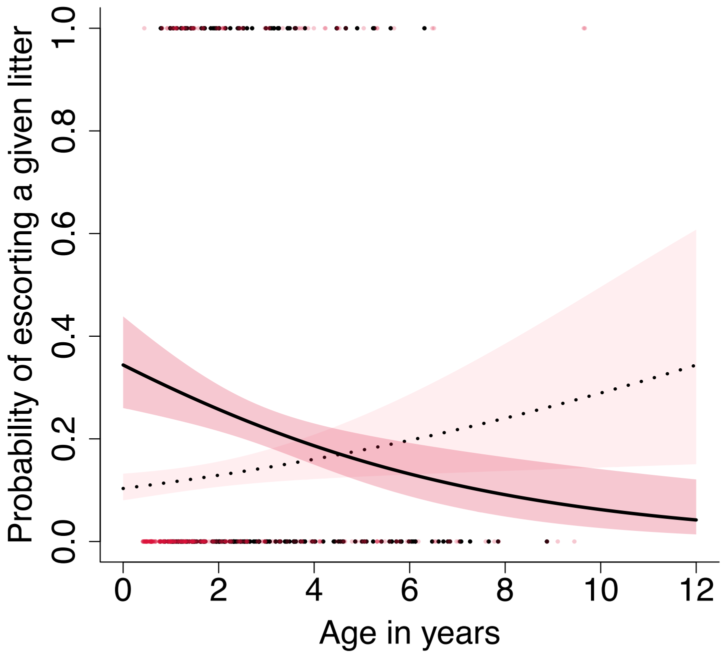

(B)

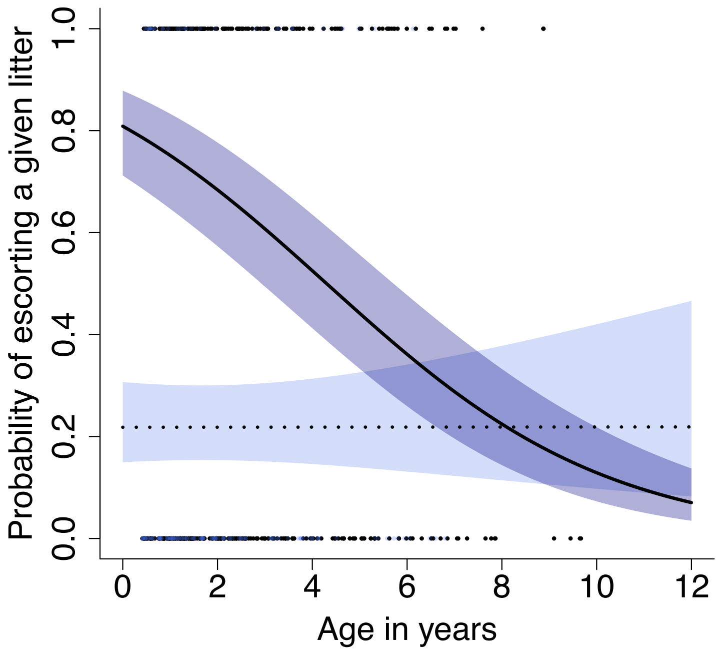

Figure S2.

Escorting effort (proportion of sessions escorting in a given litter) increased with increasing relatedness to adults in the group in males (blue) but not in females (red). Lines and shaded area represent model predictions ± SE from binomial GLMMs, after controlling for random effects of individual, litter and social group. For full results see Table S1B.

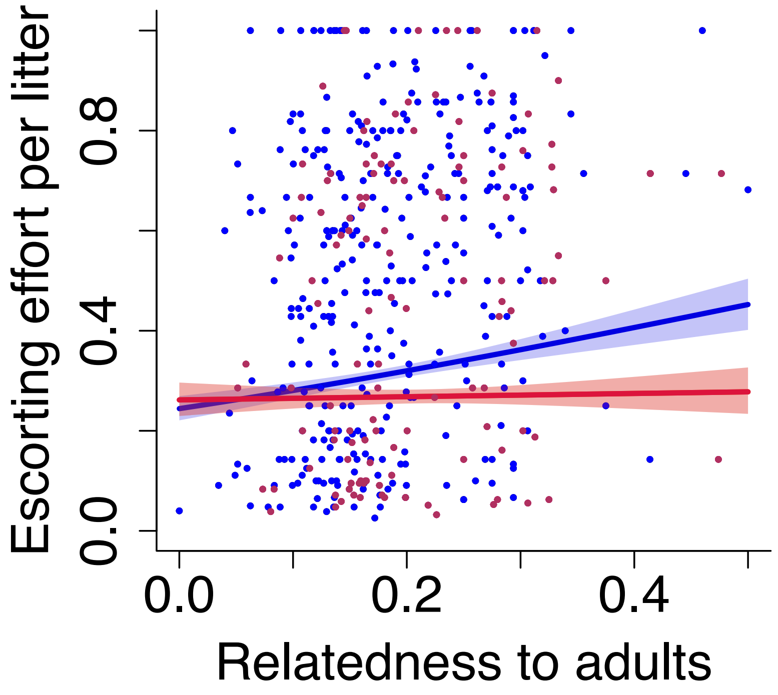

Supplement: Supplementary Information for Vitikainen et al. 2017, pup escorting in the banded mongoose [file rspb20162384supp1.pdf]
